# Autonomic dysregulation in long-term patients suffering from Post-COVID-19 Syndrome assessed by heart rate variability

**DOI:** 10.1038/s41598-023-42615-y

**Published:** 2023-09-22

**Authors:** Frank C. Mooren, Irina Böckelmann, Melina Waranski, Mona Kotewitsch, Marc Teschler, Hendrik Schäfer, Boris Schmitz

**Affiliations:** 1 Department of Rehabilitation Sciences, Faculty of Health, University of Witten/Herdecke, Witten, Germany; 2 DRV Clinic Königsfeld, Center for Medical Rehabilitation, Holthauser Talstraße 2, 58256 Ennepetal, Germany; 3 Occupational Medicine, Faculty of Medicine, Otto-Von-Guericke University, Magdeburg, Germany

**Keywords:** Diseases, Biomarkers

## Abstract

Post-COVID-19 Syndrome (PCS) is a condition with multiple symptoms partly related to dysregulation of the autonomic nerve system. Assessment of heart rate variability (HRV) using 24 h Holter-ECG may serve as a surrogate to characterize cardiac autonomic activity. A prospective study including 103 PCS patients (time after infection = 252 days, age = 49.0 ± 11.3 years, 45.7% women) was performed and patients underwent detailed clinical screening, cardiopulmonary exercise testing, and 24 h Holter monitoring. Data of PCS patients was compared to 103 CAD patients and a healthy control group (n = 90). After correction for age and sex, frequency-related variables differed in PCS patients compared to controls including LF/HFpower, LF/HFnu, and LF/HF ratio (24 h; p ≤ 0.001). By contrast, these variables were largely comparable between PCS and CAD patients, while sympathetic activation was highest in PCS patients during the 24 h period. Overall, PCS patients showed disturbed diurnal adjustment of HRV, with impaired parasympathetic activity at night. Patients hospitalized during acute infection showed an even more pronounced overactivation of sympathetic activity compared to patients who underwent ambulant care. Our data demonstrate persistent HRV alterations in PCS patients with long-term symptom duration, suggesting a sustained impairment of sympathovagal balance. Moreover, sympathetic overstimulation and diminished parasympathetic response in long-term PCS patients are comparable to findings in CAD patients. Whether HRV variables have a prognostic value in PCS and/or might serve as biomarkers indicating a successful interventional approach warrants further longitudinal studies.

## Introduction

Post-COVID-19 Syndrome (PCS) occurs as a sequela after acute infection with the SARS-CoV-2 virus (COVID-19 infection). PCS is defined as persistent symptoms over a period of 12 weeks from infection and/or the appearance of new symptoms in this period^1^. PCS can be described as a multisystem disorder with the most common symptoms include (chronic) fatigue, cognitive impairment (memory/brain dysfunction, impaired concentration, also known as brain fog), decreased physical performance, muscular weakness and pain, dyspnea, and mental and psychological distress in the sense of a post-traumatic stress reaction^2–5^. PCS can occur after a severe as well as mild or moderate course of acute infection, however individual risk factors of PCS are currently controversially discussed^1,4,6,7^. Estimates on incidence vary also depending on the population, the number/severity of symptoms considered as well as the virus variant present^4,8^. While the majority of affected patients experience a gradual healing process without targeted treatment, the need for effective medical rehabilitation is high for patients with persistent PCS^1,4^.

PCS is to some extend characterized by diagnostic vagueness as the symptomatology is complex and, due to the lack of diagnostics, not always distinct^2,3^. It has been suggested that PCS signs and symptoms may be linked to a disruption of the autonomic nervous system associated with increased sympathetic nerve activity^9–11^. While the main mechanism leading to these observations are still a matter of ongoing research, it has been reported that SARS-CoV-2 shares features of known neurotrophic viruses which cause dysautonomia through dysregulation of central and peripheral circuit of the autonomic nervous system through direct or indirect routes including retrograde axonal transport via the olfactory nerve or the enteric nervous system^10–12^. In addition, acute SARS-CoV-2 infection induces stress causing (excessive) release of pro-inflammatory cytokines such as IL-6, -2R, -8, and TNF-α, and neuro-hormonal (over)stimulation and it has been suggested that sympathetic activation in COVID-19 patients may be linked to hypoxia, immunological factors, dysregulation of the angiotensin converting enzyme axis, and emotional distress^10–13^. As a result, persistent or secondary autonomic nervous dysfunction may occur, which has been suggested to add to PCS-specific symptoms including fatigue even though the precise association is unclear^10^. Studies have used heart rate variability (HRV) to assess autonomic dysregulation in patients with acute COVID-19 infection^12^ and some have investigated HRV changes in PCS patients with short to medium symptom duration^14–18^. However, autonomic dysregulation using HRV in PCS patients with prolonged symptom duration have not been reported. Thus, the general aim of this study was to investigate if autonomic dysregulation is still present in long-term PCS patients using assessment of HRV by 24 h Holter ECG. Patients with documented coronary artery disease (CAD) were used to compare HRV alterations in PCS to a group of patients with a severe chronic cardiovascular disease. More specifically, we studied the association of HRV measures with several clinical characteristics of PCS patients such as physical fitness, infection history, clinical symptomatology, and laboratory parameters to obtain insights on the possible pathophysiological background.

## Methods

### Study populations

#### PCS patients

A prospective observational cohort study of PCS patients referred to Clinic Königsfeld, center for medical rehabilitation was performed between Mai 2021 and April 2022. Inclusion criteria were a history of (at least one) COVID-19 infection (positive PCR test at the time of infection), and ongoing or newly expressed performance deficits lasting for at least 3 months prior to recruitment. In total, 103 PCS patients were included. Performance deficits were documented according to the recent consensus statement, with the cluster of lead symptoms including fatigue/exercise intolerance, shortness of breath, and cognitive dysfunction impairing activity of daily living and everyday functioning^5^. A detailed clinical workup was performed, and history of comorbidities and current medication were documented.

#### Control groups

A group of 103 female and male patients with a diagnosis of CAD was included for comparison (Table 1). Patients had been enrolled as part of a prospective cohort study on the effectivity of medical rehabilitation. CAD patients after acute myocardial infarction and/or reperfusion via percutaneous transluminal coronary angioplasty (PTCA) and/or coronary artery bypass graft (CABG) were included without further selection (Supplementary Table S1). Detailed clinical workup, medication, 24 h Holter ECG and CPET was available for this group. Furthermore, a historic control group of 90 healthy male and female participants was included for comparison of HRV data (Table 1). Recruitment and characteristics have been described in detail elsewhere^19^.

**Table 1:** Anthropometric data of patients with Post-COVID-19 Syndrome (PCS) and control groups.

	PCS (n = 103)	CAD (n = 103)	Ctrl (n = 90)	p-value
Age, years	49.0 ± 11.3	54.9 ± 7.0	43.7 ± 10.7	< 0.0001^#^
Sex, n (%)				< 0.0001^§^
Female	44 (45.7)	13 (12.6)	12 (13.3)	
Male	59 (57.3)	90 (87.4)	78 (86.7)	
Height, cm	173.5 ± 9.4	176.7 ± 8.6	169.1 ± 8.6	< 0.0001^#^
Weight, kg	93.1 ± 24.0	92.8 ± 17.2	74.6 ± 16.1	< 0.0001*
BMI, kg*m^−2^	30.8 ± 6.7	29.6 ± 4.5	26.1 ± 5.4	< 0.0001*

Data presented as mean ± SD or n (%). Between-group comparison was performed using one-way ANOVA with Tukey's multiple comparisons test or Kruskal–Wallis test. ^#^Significantly different for PCS vs. CAD, PCS vs. CG and CAD vs. CG; *Significantly different for PCS vs. CG and CAD vs. CG; ^§^Significantly different for CG vs. PCS and CAD. PCS, Post-Covid Syndrome patients; CAD, Coronary Artery Disease patients. Crtl, healthy control group.

### Ethical approval

The study conformed to the Declaration of Helsinki and was approved by respective local ethical review committees (Ethik-Kommission Universität Witten/Herdecke; reference number 159/2021 and 115/2020 for PCS and CAD). Analysis of HRV in healthy controls was approved by the ethics committee of Otto-von-Guericke-University Magdeburg (reference number 139/12). Written informed consent was obtained from all participants.

### Assessment of perceived disease burden, functional status, and fatigue

Disease burden and functional impact on daily live including fatigue was assessed at enrollment by validated questionnaires as follows. The Multidimensional fatigue inventory (MFI-20) was used to assess fatigue in PCS as described^20^. The MFI-20 provides an overall score as well as two subscales on physical fatigue and mental fatigue. The scores range from 0–100, with higher scores indicating higher levels of fatigue. Health-related quality-of-life was assessed using the SF-36 questionnaire which includes eight health concepts: physical functioning, physical role, bodily pain, general health, vitality, social functioning, emotional role, and mental health. The SF-36 provides two scores, a Physical Component Score (PCS) and a Mental Component Score (MCS) ranging between 0 and 100, with higher scores indicating a more favorable functional status^21^. The Hospital anxiety depressions scale (HADS) was applied to assess anxiety and depression severities with subscales graded as follows: 0–7 = ‘normal’, 8–10 = ‘mild’, 11–14 = ‘moderate’, and 15–21 = ‘severe’. The WHO-5 questionnaire was used to evaluate the general level of well-being. The score ranges from 0 to 25, higher scores indicating greater wellbeing^22^. Work ability was measured using the Work Ability Index questionnaire, which includes the following subscales: present working capacity, ability to work concerning the job requirements; diagnosed pathologies; reduction in working capacity due to illness; sick leave over the past 12 months; personal expectations of one’s work skills two years onwards; psychological conditions/resources^23^. The WAI score may be rated: low (7–27), moderate (28–36), good (37–43), or excellent (44–49).

### Cardiopulmonary exercise testing (CPET)

Symptom-limited ergometer testing with continuous breath-by-breath respiratory gas exchange analysis was performed according to manufacturer’s instructions (Ergostic, Amedtech, Aue, Germany). Expiratory flow measurements were performed by a mass flow sensor, calibrated with a gas mixture of known concentration before each test. Physical fitness of PCS and CAD patients was determined during an initial clinical stress ECG and an adapted ramp protocol was chosen according to the initial stress ECG results for spiroergometry: 1. low performance (< 100 W): start at 20 W, increase by 15 W/2 min; 2. medium performance (100–125 W): start at 20 W, increase by 20 W/2 min; 3. moderate performance (> 125 W): start at 25 W, increase 25 W/2 min. Patients were instructed to reach a rating of perceived exertion of ≥ 8 on the 0–10 Borg Scale during the test. Recorded variables included heart rate (HR), blood pressure, oxygen consumption (VO_2_), carbon dioxide production (VCO_2_) and minute ventilation (VE). Peak VO_2_ was defined as the maximal oxygen uptake reported as a percentage of reference (predicted value considering sex, age, and body surface area) for comparability. VO_2_ at the anaerobic threshold (AT; first ventilatory threshold [VT1]) was identified using both V-slope method and the ventilatory equivalent method (VE/VO_2_) The oxygen pulse was calculated through the VO_2_/HR ratio. Breathing reserve represents the ratio between VE during exercise and maximum voluntary ventilation at rest.

### Assessment of heart rate variability (HRV)

Using an identical sampling rate of 1000 Hz, Holter ECG systems were used to assess HRV over at least 24 h in PCS patients and CAD patients (DMS300-4L, DM systems, Beijing, China) as well as in healthy controls (MT-101, Schiller AG, Schweiz). ECG data was imported into CardioScan 12.0 (MTM Multitechmed GmbH, Hünfelden-Dauborn, Germany) or MT-200 Holter_ECG 2.54 (Schiller AG). Data was screened and edited for artifacts and HRV values were calculated for consultation. Only ECGs with a minimum recording length of 22 h (79,200 s) were used for analyses. Out of 128 PCS patients assessed, data of 103 patents was eligible for analysis with Holter recordings > 22 h, 25 patients had shorter recordings due to detachment of electrodes or lower compliance. After the NN intervals were exported, HRV analysis was performed with Kubios HRV 2.0 (Biomedical Analysis and Medical Imaging Group, University of Kuopio, Finland) with artifact correction (settings: “custom” and “0.3”). The automated recognition of regular rhythm and artifacts of the software was checked manually, and ECGs entered statistical analyses only if the rate of sinus rhythm was higher than 90%, regardless of the cause (aberrant rhythms or artifacts). The following variables were extracted for analyses as described^19,24^: time domain variables (NN intervals, SDNN, standard deviation of all NN intervals; SDNN-Index, mean value of the standard deviations of the average NN intervals of all 5-min segments of a measurement; SDANN, standard deviation of the average NN intervals of all 5-min segments of a measurement; RMSSD, square root of the mean of the sum of the squares of differences between adjacent NN intervals; pNN50, NN50 divided by the total number of NN intervals; triangular-Index, integral of the NN interval histogram divided by the height of the histogram). Frequency domain variables (HF, average energy density in the high-frequency band [i.e., between 0.15 and 0.4 Hz of all 5-min-calculation windows]; LF, average energy density in the LF low-frequency band [i.e., between 0.04 and 0.15 Hz of all 5-min-calculation windows]; HFnu, normalized HF [HF/(total power − VLF) * 100]; LFnu, normalized LF [LF/(total power − VLF) * 100]; HF power [absolute power of the HF band]; LF power [absolute power of the LF band]). Nonlinear variables as defined by the analysis of Poincaré maps, a scatter plot of inter-beat intervals as a function of previous inter-beat intervals (SD1, the standard deviation of Poincaré plot perpendicular to the line-of-identity; SD2, the standard deviation of the Poincaré plot along the line-of-identity; VAI, the angular dispersion of scatter points). A detailed description of 24 h HRV nonlinear analysis has been described elsewhere^25^.

### Laboratory parameters

Blood samples were taken on the day of hospital admittance and were analyzed the same day at an external certified laboratory (accredited for ISO 17025 and 15189). In brief, analyses included standard cell populations, HbA1c, C-reactive protein, creatinine, urea, uric acid, lipids, and liver enzymes.

### Statistical analysis

Data was analysed using SPSS (V.28, IBM, Armonk, NY, USA). Constant variables are expressed as mean ± standard deviation (SD) or median and interquartile range (IQR) as indicated. Categorical variables are presented as n (%). Non-normal distribution was tested using skewness and kurtosis. Differences between two groups were analysed using unpaired two-sided t-test or Mann–Whitney U test in case of non-normal distribution. Chi-square test was used for categorical variables. Differences of HRV variables were analyzed using general linear model (GLM) with factors of group (PCS, CAD, control), sex, and age. Post hoc between-group comparisons were performed by one-way ANOVA and Bonferroni’s correction or Kruskal–Wallis test. Only significantly different HRV variables of the group comparison were considered for subsequent subgroup analysis. Subgroups were defined by (A) the median of days after acute infection, (B) whether subjects were hospitalized, and (C) the presence of lead symptoms, where multiple symptoms were diagnosed. Corrections for age and sex were performed where indicated. For estimation of effect sizes, partial η^2^ was used with η^2^ < 0.06 indicating a small, η^2^ = 0.06 to 0.14 indicating a medium and η^2^ > 0.14 indicating a large effect. Significance was accepted at p < 0.05.

## Results

### Patients’ characteristics

PCS patients’ mean age was 49.0 ± 11.3 years, 45.7% were women (Table 1). The median time interval between the (first) acute COVID-19 infection and start of medical rehabilitation (i. e. time of examination) was 252 (IQR 166–310) days. During acute infection, 31% had been hospitalized (38% with need for ventilation), 69% had received ambulant care or acute care at home. Patients reported to the rehabilitation center with a combination of PCS-specific lead symptoms^5^ including limited exercise tolerance/fatigue (n = 85, 82.5%), shortness of breath/exercise-induced dyspnea (n = 82, 79.6%) and cognitive dysfunction (n = 58, 56.3%). Eighty-eight patients (85.4%) presented with at least 2 lead symptoms.

### Patients’ risk factors at enrollment

Mean BMI of PCS patients was 30.8 ± 6.7 kg/m^2^, 27.2% were obese (class I or higher), 8.7% were ever smoker (Table 1, Supplementary Table S1). Structural abnormalities of the heart were detected in 17.7% of patients who underwent echocardiography, 4 (3.9%) had CAD with one or two vessel disease (Supplementary Table S1). Arterial hypertension was diagnosed in 54.4% of PCS patients, 14 (13.6%) had type 2 diabetes mellitus. Nine patients had been treated for pulmonary embolism (8.7%). Of note, a high number of patients (62.1%) reported musculoskeletal/connective tissue problems, which was significantly higher compared to the older group of CAD patients (34%, p < 0.001). A complete overview of secondary diagnoses and medications is given in Supplementary Table S1. Standard laboratory did not show any deviations from reference in PCS patients. In comparison, a lower level of leukocytes in PCS patients compared to CAD patients was seen (6.8 ± 1.8 vs. 8.3 ± 2.0 n/nl, p < 0.001), while CRP was not elevated and comparable to levels in CAD patients (Supplementary Table S1). Blood lipids except for triglycerides were all significantly lower in CAD patients (p < 0.001), reflecting the use of cholesterol-lowering therapy. A relevant number of PCS patients (17.5%) were diagnosed with depressive/adjustment disorders and 14.6% were treated with antidepressants. Median HADS depression score was 7 (i. e. unremarkable) and significantly higher compared to CAD patients (score = 4, p < 0.001) (Table 2). HADS anxiety score was not elevated and comparable to CAD patients, while depression score was significantly elevated (p < 0.001; Table 2). For HADS anxiety and HADS depression score, 19.4% and 23% of PCS patients had values > 10, respectively, indicating at least moderate psychological distress.

**Table 2:** Disease perception and patient reported outcomes.

	PCS	CAD	p-value
Multidimensional fatigue inventory (MFI-20)			
Overall score	71.0 ± 13.6	56.0 ± 14.6	< 0.001
Physical fatigue	77.7 ± 16.7	65.7 ± 18.5	< 0.001
Mental fatigue	65.1 ± 21.7	49.0 ± 17.9	< 0.001
Workability Index (WAI)	22.4 ± 8.6	28.4 ± 8.8	< 0.001
Max. incapacity for work last 12 months^§^	99 (365)	24 (365)	0.004
SF-36 health-related quality of life			
Physical component score (PCS)	30.7 ± 7.8	37.4 ± 11.1	< 0.001
Mental component score (MCS)	36.2 ± 12.2	42.3 ± 10.9	0.002
Wellbeing (WHO-5)	8 (24)	10 (22)	0.039
Hospital anxiety and depression scale (HADS)			
Anxiety	6 (20)	5 (15)	0.263
Depression	7 (21)	4 (15)	< 0.001

Data is presented as mean ± SD or median (range). Between-group comparison was performed using unpaired two-sided t-test or Mann–Whitney U test. MFI-20: range 0–100 (higher = greater fatigue); WAI: range 7–49 (higher = improved work ability); SF-36: range 0–100 (higher = greater quality of life); WHO-5: range 0–25 (higher = greater wellbeing); HADS: range 0–21 (higher = greater anxiety/depression). ^§^Patient-reported days being off work because of illness (WAI item; the maximal number of days was used to calculate the group mean). PCS, Post-Covid Syndrome patients; CAD, Coronary Artery Disease patients.

### Disease perception and patient reported outcomes

To assess patients’ disease perception and the impact of PCS on patients’ daily life, different standardized questionnaires were used (Table 2). To quantify and differentiate the extend of fatigue, the multidimensional fatigue inventory (MFI-20) was used. Results indicated that PCS patients had significantly higher levels of overall fatigue compared to CAD patients (p < 0.001) resulting from higher levels of physical as well as mental fatigue (both p < 0.001 vs. CAD). PCS patients’ general wellbeing (WHO-5 score) was lower compared to CAD patients (p < 0.001) and health-related quality of life was significantly reduced, indicated by lower Physical and Mental Component Score of the SF-36 questionnaire (p ≤ 0.002 vs. CAD). Assessment of Workability Index as a marker of disease impact on current and future workability indicated lower values for PCS patients compared to CAD patients (p < 0.001) and revealed that the median maximal incapacity for work during the last 12 months was 99 days for the PCS patient group and only 24 days for the CAD patient group (p = 0.004). Self-estimation of own work ability in the next 2 years revealed that only 34% of PCS patients estimated to be able to return to work within the next 12 months (40% did not know, 9% estimated to be unable to work) (Data not shown).

### Cardiopulmonary exercise testing (CPET)

Patients underwent a routine CPET within three days after referral to rehabilitation to assess the impairment of physical fitness. Variables depending on age, sex and body surface area were analyzed as percent of reference (i. e. percent predicted) for comparability to CAD patients. Overall, results indicated significantly reduced physical fitness of PCS patients compared to age- and sex-matched reference and a level comparable to the ~ 6 years older group of CAD patients (Supplementary Table S2). PCS patients’ pre-testing HR was significantly elevated, while the O_2_ pulse was reduced indicating lowered oxygen consumed per heartbeat. PCS patients reached the first ventilatory threshold 1 (VT1) at a lower relative workload with a lower relative O_2_ pulse compared to CAD patients (p ≤ 0.017). Mean relative peak exercise O_2_ uptake (VO_2peak_) in PCS patients was reduced by almost 30% and was comparable to the reduced VO_2peak_ of CAD patients (PCS, 72.7 ± 16.8% vs. CAD, 75.5 ± 16.0%, p = 0.290). Pulmonary variables including ventilatory equivalents for O_2_ and CO_2_, tidal volume, breathing frequency and breathing reserve were not different between both patient groups.

### Heart rate variability

#### General observation

HRV was assessed using 24 h Holter and compared to CAD patients and a historic healthy control sample. ANCOVA with age and sex as covariates revealed a set of HRV variables significantly altered in PCS patients (Table 3). For time-dependent variables, significant differences during the day period were found for SDNN and rMSSD which were elevated in PCS patients compared to healthy controls (p ≤ 0.003) and were comparable to CAD patients. While SDNN was also elevated in PCS patients at night, it differed compared to CAD patients (p = 0.014) but was comparable to controls. Moreover, pNN50 was reduced in PCS patients at night compared to controls. NN intervals were overall shorter in PCS patients, with a significant difference compared to CAD patients and controls during the night period (p < 0.001). Several frequency-related variables were detected to differ significantly in PCS patients compared to controls including LFpower, LFnu, HFpower, HFnu, and LF/HF ratio (p ≤ 0.001). Compared to controls, the day-night shift of HFnu and LFnu was abolished, driven by a missing increase of parasympathetic activity during the night (p < 0.001 vs. ctrl). Of note, these variables did not differ between PCS and CAD patients. Moreover, sympathicus activation as reflected by LF/HF ratio and percentage of sympathicus was highest in PCS patients compared to controls and CAD patients during the 24 h period (p ≤ 0.001). With regard to non-linear variables, VAI was significantly reduced with PCS, compared to both, CAD patients and controls (p ≤ 0.001), while SD1 was comparable to controls but lower than in CAD patients (p = 0.016).

**Table 3:** Heart rate variability (HRV) by group over 24 h, day, and night period.

(A)	Group						Adjusted model			Sex		Age		Group	
	PCS (n = 103)		CAD (n = 103)		Ctrl (n = 90)		F	p	η^2^	p	η^2^	p	η^2^	p	η^2^
	M	SD	M	SD	M	SD									
Time-related variables (24 h)															
NN interval, ms	797.2	93.7	858.9	121.9	797.6	81.5	9.378	** < 0.001**	0.115	** < 0.001**	0.039	0.569	0.001	**0.009**^**§**^^,^*	0.032
SDNN, ms	154.7	39.9	147.0	49.9	146.9	33.3	7.879	** < 0.001**	0.098	** < 0.001**	0.057	**0.001**	0.40	0.197	0.011
SDANN, ms	139.4	39.0	130.7	46.1	130.0	32.3	6.355	** < 0.001**	0.080	** < 0.001**	0.050	**0.010**	0.023	0.105	0.015
SDNN Index, ms	59.1	18.0	53.8	19.5	62.6	15.0	18.929	** < 0.001**	0.206	** < 0.001**	0.054	** < 0.001**	0.135	0.013*	0.029
rMSSD, ms	42.2	20.2	36.4	20.4	36.4	14.1	8.311	** < 0.001**	0.103	**0.002**	0.032	** < 0.001**	0.055	**0.046**	0.021
pNN50, %	9.1	8.0	9.3	11.1	11.7	8.5	13.719	** < 0.001**	0.159	**0.011**	0.022	** < 0.001**	0.130	0.268	0.009
Triangular Index	39.8	11.3	37.3	14.0	40.8	9.1	6.844	** < 0.001**	0.086	0.062	0.012	** < 0.001**	0.063	0.437	0.006
Frequency-related variables (24 h)															
LF power, ms^2^	740.1	426.7	597.8	453.6	978.4	576.0	35.804	** < 0.001**	0.330	** < 0.001**	0.069	** < 0.001**	0.222	** < 0.001**^#,^*	0.064
HF power, ms^2^	271.5	236.2	247.2	275.8	541.7	523.2	28.063	** < 0.001**	0.278	0.195	0.006	** < 0.001**	0.180	** < 0.001**^#,^*	0.054
LF/HF ratio	3.7	2.2	3.2	1.9	2.4	1.2	10.494	** < 0.001**	0.126	**0.001**	0.037	**0.012**	0.021	**0.001**^**§,#**^	0.045
LFnu	74.5	9.9	71.4	12.1	67.3	10.5	8.589	** < 0.001**	0.106	**0.017**	0.019	**0.012**	0.021	**0.001**^**§,#**^	0.046
HFnu	25.5	9.8	28.6	12.1	32.7	10.4	8.521	** < 0.001**	0.105	**0.017**	0.019	**0.012**	0.021	**0.001**^**§,#**^	0.046
Non-linear variables (24 h)															
SD1, ms	32.4	43.6	57.5	130.4	29.5	21.2	2.185	0.071	0.029	0.315	0.003	0.422	0.002	**0.016**^**§**^,*	0.028
SD2, ms	219.5	61.1	271.7	457.6	205.3	47.3	1.007	0.404	0.014	0.442	0.002	0.633	0.001	0.476	0.005
VAI	0.558	0.324	0.786	1.506	1.228	0.1323	7.845	** < 0.001**	0.097	0.084	0.010	0.211	0.005	**0.001**^**§,#**^	0.052

(B)	Group						Adjusted model			Sex		Age		Group	
	PCS (n = 103)		CAD (n = 103)		Ctrl (n = 90)		F	p	η^2^	p	η^2^	p	η^2^	p	η^2^
	M	SD	M	SD	M	SD									
Time-related variables (6 h day)															
NN interval, ms	735.0	86.7	796.8	113.2	757.1	103.6	7.165	** < 0.001**	0.090	**0.006**	0.026	0.299	0.004	**0.002**^**§**^^,^*	0.043
SDNN, ms	124.1	32.5	121.3	43.6	92.5	21.7	15.741	** < 0.001**	0.178	**0.019**	0.019	**0.010**	0.023	** < 0.001**^#,^*	0.0936
rMSSD, ms	40.2	21.2	35.1	21.5	28.8	11.3	7.338	** < 0.001**	0.092	**0.010**	0.023	**0.031**	0.016	**0.003**^**#**^	0.040
pNN50, %	7.4	7.1	8.1	11.6	8.1	7.8	5.364	** < 0.001**	0.069	0.066	0.012	** < 0.001**	0.058	0.702	0.002
Frequency-related variables (6 h day)															
LF power, ms^2^	643.0	414.7	477.8	416.3	959.2	578.2	43.666	** < 0.001**	0.375	** < 0.001**	0.046	** < 0.001**	0.242	** < 0.001**^#,^*	0.090
HF power, ms^2^	221.9	201.9	218.2	281.1	346.7	340.5	13.736	0.079	0.159	0.160	0.007	** < 0.001**	0.117	0.073	0.018
LF/HF ratio	3.9	2.3	3.0	2.0	4.0	2.7	3.933	** < 0.001**	0.051	**0.024**	0.017	0.409	0.002	**0.001**^**§**^^,^*	0.051
LFnu	75.4	9.7	69.6	13.5	75.7	9.6	5.185	** < 0.001**	0.067	0.187	0.006	0.995	0.000	** < 0.001**^**§**^^,^*	0.058
HFnu	24.6	9.7	30.4	13.5	24.3	9.6	5.185	** < 0.001**	0.067	0.187	0.006	0.995	0.000	** < 0.001**^**§**^^,^*	0.058

(C)	Group						Adjusted model			Sex		Age		Group	
	PCS (n = 103)		CAD (n = 103)		Ctrl (n = 90)		F	p	η^2^	p	η^2^	p	η^2^	p	η^2^
	M	SD	M	SD	M	SD									
Time-related variables (6 h night)															
NN interval, ms	909.4	127.2	963.2	152.8	959.5	109.2	7.126	** < 0.001**	0.090	** < 0.001**	0.057	0.819	0.001	** < 0.001**^**§,#**^	0.056
SDNN, ms	103.9	33.3	93.9	31.0	96.1	24.3	14.442	** < 0.001**	0.166	** < 0.001**	0.081	** < 0.001**	0.082	**0.019**^**§**^	0.027
rMSSD, ms	45.9	24.4	38.6	23.3	47.7	23.0	12.781	** < 0.001**	0.149	**0.011**	0.022	** < 0.001**	0.110	0.168	0.012
pNN50, %	13.8	13.3	12.7	13.9	20.7	16.0	22.333	** < 0.001**	0.235	**0.005**	0.026	** < 0.001**	0.173	**0.014**^**#**^	0.029
Frequency-related variables (6 h night)															
LF power, ms^2^	923.0	594.2	812.2	652.1	1162.7	863.3	19.185	** < 0.001**	0.209	** < 0.001**	0.076	** < 0.001**	0.119	**0.001**^#,^*	0.045
HF power, ms^2^	366.5	355.8	306.1	367.9	862.7	888.8	30.715	** < 0.001**	0.297	0.184	0.006	** < 0.001**	0.166	** < 0.001**^#,^*	0.077
LF/HF ratio	3.8	2.7	3.9	3.6	1.9	1.2	10.9	** < 0.001**	0.13	0.059	0.012	**0.006**	0.0126	**0.009**^**#**^	0.031
LFnu	73.5	12.4	73.3	12.5	61.3	13.4	22.188	** < 0.001**	0.234	**0.004**	0.028	** < 0.001**	0.058	** < 0.001**^**#**^	0.060
HFnu	26.5	12.4	26.7	12.5	38.7	13.4	22.113	** < 0.001**	0.233	**0.004**	0.028	** < 0.001**	0.058	** < 0.001**^**#**^	0.059

Data is presented as mean ± SD. Differences of HRV variables were analyzed using general linear model (GLM) with factors of group (PCS, CAD, control) and sex and age. Post hoc between-group comparisons were performed by one-way ANOVA and Bonferroni’s correction or Kruskal–Wallis test. PCS, Post-Covid Syndrome; CAD, Coronary Artery Disease.

^§^Significant for PC vs. CAD; ^#^significant for PC vs. control; *significant for CAD vs. control (Ctrl).

Significant values are in bold.

### PCS-specific HRV

To investigate whether identified HRV variables were PCS-specific, we analyzed if HRV was affected by severity of the acute COVID-19 infection and duration/symptoms of PCS, respectively, as indicated by (1) need for hospitalization (2) days after acute infection and (3) type and number of lead symptoms (Table 4). The analysis suggested that longer persistence of PCS symptoms significantly affected predominantly frequency-related variables with a decline of sympathetic stimulation. In contrast, the need for hospitalization (after correction for age) also affected time-related HRV variables. Comparison revealed that PCS patients who had undergone more severe acute infections presented with significantly lower values for SDNN (index) and rMSSD compared to patients with milder acute infections (p ≤ 0.007) and showed higher sympathetic activity in the frequency related variables. Patients with longer persistence of PCS symptoms showed a significantly lower LF/HF ratio during the day (p = 0.014) and overall higher parasympathetic activation (p = 0.006). While the quantity of concurrent main symptoms as well as presence of dyspnea was not significantly associated with HRV changes, diagnosis of physical impairment as a lead symptom was linked to HRV alterations in that patient with this lead symptom showed higher SDNN values (6 h day; p < 0.0001). This finding was further validated using ergospirometric data which provided evidence that differences in HRV were pronounced when patients were grouped by their relative impairment of physical exercise capacity (Table 5). Consistently, patients with lower aerobic capacity showed a higher LF/HF ratio (p ≤ 0.04) and LFnu (p ≤ 0.01) during the 24 h period, while HFnu was reduced (p ≤ 0.01), indicating a decreased parasympathetic activation. During the nighttime period, lower aerobic capacity was associated with enhanced SDNN (p ≤ 0.01) and LFnu (p ≤ 0.02), while HFnu was reduced (p ≤ 0.02). In contrast, patients’ disease perception as indicated by the applied questionnaires did not correlate significantly with altered HRV parameters.

**Table 4:** Heart rate variability by time after COVID-19 acute infection, acute infection severity, and current symptomology.

	Days after infection		*p*	Hospitalization		*p*	Main Symptoms								*p*
							Physical impairment		*p*	Dyspnea		*p*	Cognitive impairment		
	< 252	> 252		No	Yes		No	Yes		No	Yes		No	Yes	
Time-related variables (24 h)															
NN interval, ms	810 ± 112	787 ± 76	0.222	817 ± 88	755 ± 97	**0.003**	765 ± 88	806 ± 95	0.096	745 ± 106	800 ± 92	0.838	787 ± 78	807 ± 105	0.263
SDNN Index, ms	60.1 ± 20.2	57 ± 16.4	0.302	62.4 ± 1.9	51.2 ± 2.9	**0.002**	52.1 ± 15.3	60.3 ± 18.4	0.056	58.1 ± 18	61.8 ± 18.9	0.430	58.1 ± 16.8	59.5 ± 19.2	0.697
rMSSD, ms	41.5 ± 22.2	42.5 ± 18.9	0.804	45.9 ± 2.3	34.2 ± 3.5	**0.007**	37.8 ± 19.8	43.2 ± 20.3	0.302	42.2 ± 26.2	42.3 ± 18.6	0.988	39.1 ± 15.4	44.7 ± 23.1	0.142
Frequency-related variables (24 h)															
LF power, ms^2^	793 ± 446	697 ± 412	0.272	793 ± 44.4	633 ± 66.8	0.051	606 ± 327	772 ± 438	0.076	800 ± 432	729 ± 423	0.504	748 ± 440	739 ± 414	0.914
HF power, ms^2^	256 ± 224	291 ± 253	0.462	317 ± 23.6	170 ± 35.5	**0.001**	194 ± 171	287 ± 246	0.063	288 ± 223	267 ± 241	0.703	243 ± 222	292 ± 246	0.285
LF/HF ratio	4.2 ± 2.3	3.1 ± 1.9	**0.014**	3.3 ± 0.25	4.4 ± 0.38	**0.025**	4.2 ± 2.8	3.6 ± 2.0	0.381	3.5 ± 1.9	3.7 ± 2.3	0.579	4.2 ± 2.3	3.3 ± 2.1	0.058
LFnu	77.2 ± 9.5	71.8 ± 9.6	**0.006**	72.5 ± 1.11	79.3 ± 1.67	**0.001**	76.6 ± 9.8	74.2 ± 9.8	0.360	74.2 ± 10.6	74.8 ± 9.7	0.816	77.2 ± 9	72.8 ± 10.1	**0.029**
HFnu	22.8 ± 9.5	28.2 ± 9.6	**0.006**	27.5 ± 1.11	20.7 ± 1.67	**0.001**	23.4 ± 9.8	25.8 ± 9.8	0.360	25.8 ± 10.6	25.2 ± 9.7	0.816	22.8 ± 9	27.2 ± 10.1	**0.029**
Non-linear variables (24 h)															
SD1, ms	25.3 ± 10.2	40.1 ± 61.8	0.101	36.2 ± 5.1	24 ± 7.8	0.200	23.6 ± 11	34.4 ± 47.8	0.065	38.4 ± 44.3	31 ± 43.8	0.496	34.2 ± 57.7	31.2 ± 28.9	0.751
VAI, °	0.6 ± 0.3	0.6 ± 0.3	0.903	0.61 ± 0.04	0.45 ± 0.06	**0.023**	0.4 ± 0.3	0.6 ± 0.3	0.108	0.6 ± 0.4	0.5 ± 0.3	0.384	0.5 ± 0.3	0.6 ± 0.3	0.363
Time-related variables (6 h day)															
NN interval, ms	742 ± 95	729 ± 80	0.507	732 ± 77	741 ± 105	0.598	711 ± 91	741 ± 86	0.173	748 ± 96	733 ± 85	0.500	735 ± 85	737 ± 89	0.931
SDNN, ms	130 ± 35	121 ± 30	0.174	131 ± 3.6	111 ± 5.4	**0.002**	105 ± 19.5	129 ± 32.9	** < 0.001**	126 ± 23.4	125 ± 34.1	0.809	126 ± 32.6	124 ± 32	0.751
rMSSD, ms	38.6 ± 22.4	41.3 ± 20.5	0.539	43.7 ± 2.5	32.6 ± 3.7	**0.015**	34.8 ± 19.1	41.4 ± 21.5	0.199	39.5 ± 26.4	40.5 ± 19.7	0.871	37.3 ± 16	42.6 ± 24.3	0.185
Frequency-related variables (6 h day)															
LF power, ms^2^	664 ± 440	634 ± 399	0.728	698 ± 40.7	534 ± 61.2	**0.030**	474 ± 268	683 ± 429	**0.011**	735 ± 459	624 ± 399	0.318	635 ± 413	655 ± 414	0.803
HF power, ms^2^	194 ± 178	250 ± 225	0.171	270 ± 222	115 ± 76	**0.001**	297 ± 301	380 ± 365	0.368	403 ± 363	357 ± 355	0.592	321 ± 316	401 ± 383	0.260
LF/HF ratio	4.5 ± 2.3	3.4 ± 2.1	**0.014**	3.7 ± 0.27	4.5 ± 0.41	0.104	4.3 ± 2.5	3.8 ± 2.3	0.440	4.0 ± 2.2	3.9 ± 2.4	0.916	4.3 ± 2.6	3.7 ± 2.1	0.213
LFnu	78.3 ± 9.3	72.9 ± 9.4	**0.006**	74.2 ± 1.1	78.6 ± 1.72	**0.038**	77.4 ± 9.8	75.2 ± 9.7	0.394	76.5 ± 9.1	75.4 ± 9.9	0.638	76.8 ± 9.8	4.7 ± 9.6	0.269
HFnu	21.7 ± 9.3	27.1 ± 9.4	**0.006**	25.8 ± 1.1	21.4 ± 1.72	**0.038**	22.6 ± 9.8	24.8 ± 9.7	0.394	23.5 ± 9.1	24.6 ± 9.9	0.638	23.2 ± 9.8	25.3 ± 9.6	0.269
Time-related variables (6 h night)															
NN interval, ms	932 ± 142	889 ± 111	0.104	939 ± 118	844 ± 127	** < 0.001**	880 ± 109	916 ± 131	0.278	900 ± 144	912 ± 124	0.712	896 ± 109	920 ± 140	0.356
SDNN, ms	111 ± 33.4	97.2 ± 31.6	**0.033**	107 ± 3.8	97.7 ± 5.7	0.168	96.9 ± 32.9	106 ± 32.9	0.296	104 ± 33.2	105 ± 33.0	0.944	109 ± 32.8	101 ± 32.8	0.173
pNN50, %	14.8 ± 13.9	12.8 ± 12.9	0.478	15.8 ± 1.4	8.9 ± 2	**0.008**	10.2 ± 9.9	14.4 ± 13.8	0.132	14.6 ± 11.4	13.5 ± 13.8	0.709	12.2 ± 11.8	14.9 ± 14.3	0.289
Frequency-related variables (6 h night)															
LF power, ms^2^	1039 ± 607	814 ± 569	0.060	975 ± 68.7	819 ± 103	0.218	863 ± 551	939 ± 601	0.604	915 ± 547	928 ± 604	0.925	962 ± 597	899 ± 590	0.592
HF power, ms^2^	373 ± 350	370 ± 373	0.970	429 ± 37	226 ± 56	**0.003**	297 ± 301	380 ± 365	0.313	403 ± 363	356 ± 355	0.599	321 ± 316	401 ± 383	0.249
LF/HF ratio	4.2 ± 2.9	3.3 ± 2.4	0.191	3.4 ± 0.3	4.8 ± 0.5	**0.014**	4.4 ± 3.5	3.7 ± 2.4	0.426	3.2 ± 2.3	4.0 ± 2.7	0.193	4.3 ± 2.5	3.4 ± 2.7	0.081
LFnu	75.5 ± 12.4	70.9 ± 12.2	0.064	71.2 ± 1.4	79 ± 2.1	**0.003**	76.2 ± 12	73 ± 12.4	0.327	70.1 ± 14.2	74.5 ± 11.8	0.210	76.8 ± 10.4	71.1 ± 13.3	**0.016**
HFnu	24.5 ± 12.4	29.1 ± 12.2	0.064	28.8 ± 1.4	21 ± 2.1	**0.003**	23.8 ± 12	27 ± 12.4	0.327	29.9 ± 14.2	25.5 ± 11.8	0.210	23.2 ± 10.4	28.9 ± 13.3	**0.016**

Data is presented as mean ± SD (or SE if indicated). P values were calculated using independent t-test, Kruskal–Wallis test or one-way ANOVA corrected for age and sex if indicated. Only significantly altered HRV variables (Table 3) were considered for this subgroup analysis. Subgroups were defined by the median of days after acute infection, whether subjects had been hospitalized during acute infection, and the presence of lead symptoms. Significant p-values were bold.

**Table 5:** Heart rate variability of patients with Post-COVID-19 Syndrome by relative impairment of physical exercise capacity.

	VO_2_, % predicted at VT1		p-value	VO_2peak_, % predicted		p-value
	≤ 49.1%	> 49.1%		≤ 72.7%	> 72.7%	
Time-related variables (24 h)						
NN interval, ms	790.33 ± 96.71	798.57 ± 88.33	0.68	805.0 ± 99.24	782.49 ± 80.59	0.24
SDNN Index, ms	62.42 ± 20.82	57.84 ± 15.64	0.13	62.17 ± 17.92	57.54 ± 18.62	0.34
rMSSD, ms	43.74 ± 22.96	42.78 ± 19.50	0.42	45.10 ± 19.46	40.71 ± 22.85	0.12
Frequency-related variables (24 h)						
LF power, ms^2^	853.24 ± 459.77	704.84 ± 396.74	0.06	841.37 ± 427.86	703.67 ± 426.24	0.10
HF power, ms^2^	292.09 ± 255.25	282.62 ± 244.56	0.43	319.35 ± 282.76	248.03 ± 193.90	0.07
LF/HF ratio	4.16 ± 2.54	3.31 ± 2.04	**0.04**	4.01 ± 2.73	3.39 ± 1.66	0.09
LFnu	76.97 ± 8.51	72.14 ± 11.11	**0.01**	75.24 ± 10.03	73.75 ± 10.27	0.25
HFnu	23.03 ± 8.51	27.86 ± 11.11	**0.01**	24.77 ± 10.03	26.25 ± 10.27	0.25
Non-linear variables (24 h)						
SD1, ms	40.47 ± 64.40	26.76 ± 14.50	0.09	34.98 ± 55.41	31.32 ± 32.85	0.36
VAI	0.63 ± 0.38	0.54 ± 0.28	0.13	0.60 ± 0.36	0.57 ± 0.31	0.39
Time-related variables (6 h day)						
NN interval, ms	738.52 ± 87.09	746.74 ± 88.22	0.66	739.69 ± 81.18	746.63 ± 93.95	0.712
SDNN, ms	125.33 ± 37.68	126.73 ± 28.04	0.42	126.02 ± 35.86	126.02 ± 29.07	0.50
rMSSD, ms	42.30 ± 23.68	39.84 ± 20.66	0.30	42.90 ± 20.42	38.51 ± 23.80	0.18
Frequency-related variables (6 h day)						
LF power, ms^2^	744.67 ± 484.12	602.91 ± 351.69	0.06	729.51 ± 429.79	608.84 ± 410.71	0.09
HF power, ms^2^	273.81 ± 299.14	220.06 ± 210.60	0.16	285.07 ± 307.35	193.83 ± 163.73	0.07
LF/HF ratio	4.26 ± 2.52	3.67 ± 2.29	0.13	4.25 ± 2.79	3.63 ± 1.80	0.11
LFnu	76.85 ± 9.64	74.16 ± 10.14	0.10	74.47 ± 11.02	75.44 ± 8.54	0.47
HFnu	23.16 ± 9.64	25.84 ± 10.14	0.10	25.54 ± 11.02	24.56 ± 8.54	0.47
Time-related variables (6 h night)						
NN interval, ms	892.05 ± 142.05	915.44 ± 111.23	0.39	905.63 ± 139.05	904.34 ± 109.97	0.961
SDNN, ms	115.05 ± 36.73	96.51 ± 27.26	**0.01**	110.48 ± 34.35	99.59 ± 31.20	0.14
pNN50, %	15.26 ± 15.01	13.64 ± 12.74	0.30	16.00 ± 15.35	12.49 ± 11.55	0.18
Frequency-related variables (6 h night)						
LF power, ms^2^	1058.33 ± 552.41	896.45 ± 649.99	0.11	1051.68 ± 576.39	883.36 ± 627.63	0.22
HF power, ms^2^	387.22 ± 373.98	392.27 ± 380.04	0.48	425.16 ± 408.78	328.29 ± 309.76	0.11
LF/HF ratio	4.28 ± 3.11	3.53 ± 2.35	0.1	4.08 ± 3.08	3.63 ± 2.26	0.44
LFnu	76.24 ± 10.45	70.70 ± 14.62	**0.02**	74.47 ± 11.98	72.20 ± 13.98	0.64
HFnu	23.76 ± 10.45	29.30 ± 14.62	**0.02**	25.54 ± 11.98	27.80 ± 13.98	0.64

Data is presented as mean ± SD. Differences of HRV variables between PCS patients with lower or higher physical fitness (determined by group mean of performance at ventilatory threshold 1 [VT1] and peak oxygen uptake [VO2peak]) were analyzed using unpaired t-test or Mann–Whitney U test.

Significant values are in bold.

## Discussion

This study investigated heart rate variability (HRV) as a marker of autonomic dysregulation in patients with Post-COVID-19 Syndrome (PCS) with a mean symptom duration of 252 days. In brief, the key findings of the current study are (1) HRV of long-term PCS patients is altered compared to healthy controls when adjusted for age and sex, indicated primarily by frequency related and non-linear HRV variable, while time domain measures were not significantly affected, (2) HRV alterations were largely comparable between PCS and CAD patients, (3) HRV alterations of PCS patients were more pronounced with acute COVID-19 infection severity as well as stronger impairment of physical exercise capacity, and (4) diurnal HRV analysis showed a disturbance of day-night autonomic activity possibly indicating an impaired regeneration during sleep. Together, these findings suggest that an imbalance of sympathovagal equilibrium which has been shown for the acute and post-acute phase of COVID-19 is still present in long term PCS patients.

PCS is a multifaceted clinical condition which in general is characterized by reduced physical and cognitive performance. According to a recent Delphi consensus, PCS condition includes, but is not limited to, lead symptoms such as fatigue, shortness of breath, and cognitive dysfunction impairing activity of daily living and everyday functioning^5^. In our cohort of patients referred to medical rehabilitation, more than 85% of the study participants reported at least two lead symptoms such as limited exercise tolerance/fatigue, shortness of breath/exercise-induced dyspnea and cognitive dysfunction. The pathophysiological mechanisms underlying the PCS-specific systemic performance decrease are a matter of ongoing investigations and may involve alterations in different tissues and functions. Alterations of hemostasiology and the microvasculature structure leading to impaired oxygen transfer at different locations, across the alveolo-capillary membrane and the erythrocyte membrane as well as entry into muscle cells have been discussed^26^. Likewise, a reduction in peak oxygen uptake (VO_2_) along with an exaggerated hyperventilatory response during cardiopulmonary exercise testing has been observed in our cohort and others^27^. There is also evidence that brain demyelination might add to long-term neurological and cognitive complications after COVID-19^28^. Several of the associated pathological processes might be triggered by a dysfunctional immune response during both the acute and the chronic phase of COVID-19. As reported from different other viral infections, there is evidence that SARS-CoV-2 may be associated with latent virus reactivation and/or autoimmune processes through disturbances of immune cell homeostasis, e.g. reduction of regulatory T cells (Treg), T cell overstimulation and exhaustion, and production of autoantibodies^29^.

Recently, dysregulation of autoantibodies against different G-protein coupled receptors have been described in PCS patients and their relevance on both positive and negative chronotropic effects has been demonstrated in cell culture experiments^30,31^. This observation might, at least in part, add to the observation that cardiac autonomic regulation is impaired during and after a COVID-19 infection resulting in a sympathetic over-activation and impaired parasympathetic activity^10^. Of note, autoantibodies against receptors involved in the autonomous nervous system have been correlated with symptom severity in PCS^31^. Related symptoms may include postural orthostatic tachycardia, chest pain, and inappropriate sinus tachycardia. Some initial studies already suggested autonomous dysregulation during the early phase after COVID-19 infection using HRV as an indicator of autonomic regulation of the cardiovascular system^14–18^. Shah and colleagues reported a significant reduction of SDNN and rMSSD in women and men recently recovered from COVID-19 (30–45 days after an acute infection) and suggested that alterations of rMSSD were inversely correlated to inflammatory markers CRP and interleukin-6^15^. Another study reported differences for frequency related and nonlinear HRV variables such as VLF band and alpha2 in a group of young males 4–6 weeks after a COVID-19 infection^16^. Aranyo et al. identified altered daytime pNN50 and SDNN as well as different frequency bands in COVID-19 patients suffering from inappropriate sinus tachycardia^17^. The longest HRV observation period after COVID-19 infection so far was approximately 3 months and reported both, time and frequency domain measures to be affected, the latter being comparable to our results^32^.

To the best of our knowledge, our study is first to provide evidence that altered HRV including sympathetic/parasympathetic dysregulation is still present in PCS patients with a mean symptom duration of 250 days. Accordingly, our data indicates a reduction in the LF band, while the LF/HF ratio was significantly enhanced in PCS patients. Of note, the latter variable has been discussed to potentially indicate the degree of sympathovagal balance^33,34^. Time domain measures, however, did not show any significant differences between PCS patients and controls during the 24 h period. These findings contrast reports in post-acute COVID-19 patients, which might be explained by either the various disease states (acute/post-acute/long-term), different kinetics/sensitivity of the various HRV parameters during the course of the disease or different HRV registration modes^32^. At least for the time course of the disease an effect on HRV parameters was identified since LF/HF ratio decreased significantly with increasing time after infection. The specificity of our findings is supported by the correlation to the severity of the acute infection i. e. the need for hospitalization. Our findings on sympathetic hyperstimulation might be explained by adrenergic auto-antibodies in patients with persistent PCS symptoms, which have been suggested to target the β2-adrenoceptor, the α1-adrenoceptor, the angiotensin II AT1-receptor, and the nociceptin-like opioid receptor^30^ and which exert chronotropic effects at least in neonatal rat cardiomyocytes in vitro^35^. Another main finding of our study is that HRV alterations including the LF and HF band were similar between patients with PCS and CAD, which is of relevance since CAD patients have a long-term chronic disease with significant cardiac and vascular manifestations. Some HRV variables such as the LF/HF ratio were even more affected in PCS patients compared to CAD patients and sympathetic activation over 24 h was highest in PCS patients. Our findings may be of relevance since HRV alterations have been established as an independent prognostic marker of (all-cause) mortality and nonfatal cardiac events in patients with different cardiovascular diseases^36–39^. If auto-antibody concentrations over time can be linked to HRV alterations also in long-term PCS patients and whether HRV alterations might serve as a predictor of morbidity or mortality of PCS patients will be the scope of future studies.

With regard to clinical symptomatology, some overlap between PCS and functional somatic disorders/syndromes such as chronic fatigue syndrome (CFS) and fibromyalgia has been suggested, and considerable comparability of HRV alterations seem to exist^40,41^. In CFS patients, a reduction in the HF band has been reported, associated with an increased LF/HF ratio, which is in accordance with HRV data of PCS patients reported here. Moreover, night-time parasympathetic activity has been reported to be reduced in CFS patients^42^, which is comparable to disturbed diurnal HRV changes observed in our PCS cohort. These observations might add to the fatigue symptomatology as it can be expected to disturb effective regeneration. Similarly, our diurnal HRV analysis further showed that during the day, time domain measures such as SDNN and rMSSD were higher than during the night despite a shorter mean NN interval. This might reflect the observed sleepiness/fatigue of PCS patients together with an increased frequency of resting periods during the day. In addition, for both PCS and CFS a relation between autonomic dysfunction as well as fatigue levels has been described^43^.

There are some limitations of this study. First, registration of HRV variables for PCS/CAD patients and controls has been performed using different devices. However, application of normalized values, should have minimized the effects. The time of HRV assessment during inpatient rehabilitation including effects of the therapeutic (exercise) program may have affected the analysis, even though programs are largely comparable for PCS and CAD patients. Since a recent meta-analysis revealed a small but significant positive effect on HRV with guideline-based CAD medication including beta-blockers^44^, HRV data of included CAD patients may have been affected by this. Last, even though PCS patients enrolled in this study were characterized by long-term symptom persistence, they were capable of participating in a medical rehabilitation program and our findings may not be transferred to PCS patents with greater symptom severity.

We conclude that in PCS patients with long-term symptom duration persisting HRV alterations exist, which indicate an impaired sympathovagal balance. PCS patients showed signs of a sympathetic overstimulation and a diminished parasympathetic response comparable to patients with CAD. In addition, the relation of these HRV anomalies to the severity of the acute COVID-19 infection supports their relevance. Whether HRV variables might have a prognostic value for PCS and/or might serve as biomarkers during a successful interventional approach warrants further longitudinal studies.

### Supplementary Information

Supplementary Table S1.

Supplementary Table S2.

## Data Availability

The datasets used in this study are available from the corresponding author upon reasonable request.
