# Supplementary material for: Autonomic dysregulation in long-term patients suffering from Post-COVID-19 Syndrome assessed by heart rate variability
Source: Sci Rep. 2023 Sep 22;13:15814. doi: 10.1038/s41598-023-42615-y (PMC10516975; doi:10.1038/s41598-023-42615-y)
Supplement: Supplementary file 1 — Supplementary Table S1. [file 41598_2023_42615_MOESM1_ESM.pdf]

Supplementary Table S1: Clinical data, medication and blood parameters

	PCS (n=103)	CAD (n=103)	p-value
**Clinical data**			
**Diseases of the circulatory system**			
**Coronary artery disease, n (%)**	4 (3.9)	103 (100)	<0.001
one vessel disease	2 (1.9)	34 (33)	-
two vessel disease	2 (1.9)	40 (38.8)	-
three vessel disease	0 (0)	29 (28.2)	-
STEMI/NSTEMI, n (%)	2 (1.9)	77 (74.8)	<0.001
**LVEF[§], %**	58.9 ± 4.5	56.3 ± 7.1	0.012
**Structural abnormalities[§], n (%)**	11 (17.7)	42 (43.4)	<0.001
Cardiac arrhythmia, n (%)	7 (6.8)	11 (10.7)	0.324
Ventricular fibrillation/ flutter	4 (3.9)	10 (9.7)	-
Paroxysmal tachycardia	3 (2.9)	1 (1)	-
Arterial hypertension, n (%)	56 (54.4)	92 (89.3)	<0.001
Pulmonary embolism, n (%)	9 (8.7)	0 (0)	0.002
**Endocrine, nutritional or metabolic diseases, n (%)**			
Obesity	28 (27.2)	26 (25.2)	0.751
Type 2 diabetes mellitus	14 (13.6)	28 (27.2)	0.015
Other	29 (28.2)	78 (75.7)	<0.001
**(Hypo/hyper)tyroidism, n (%)**	12 (11.7)	11 (10.7)	0.825
**Diseases of the digestive system, n (%)**	16 (15.5)	7 (6.8)	0.046
**Diseases of the nervous system, n (%)**	19 (18.4)	13 (12.6)	0.248
**Diseases of the respiratory system, n (%)**	40 (38.8)	10 (9.7)	<0.001
**Diseases of the musculoskeletal system/ connective tissue, n (%)**	64 (62.1)	35 (34)	<0.001
**Depressive/ adjustment disorders, n (%)**	18 (17.5)	12 (11.7)	0.236
**Diseases of the genitourinary system, n (%)**	6 (5.8)	7 (6.8)	0.774
**Migraine/ headache, n (%)**	6 (5.8)	1 (1)	0.055
**Medication**			
**ACE inhibitor**	21 (20.4)	61 (59.2)	<0.001
**Statin**	15 (14.6)	99 (96.1)	<0.001
**Beta blocker**	34 (33.0)	86 (83.5)	<0.001
**AT-II receptor blocker**	17 (16.5)	31 (30.1)	0.031
**Calcium channel blocker**	17 (16.5)	25 (24.3)	0.226
**Anticoagulant**	21 (20.4)	100 (97.1)	<0.001
**Antiarrhythmic**	0 (0)	2 (1.9)	0.498
**Diuretic**	21 (20.4)	22 (21.4)	1.0
**Glucocorticoid**	17 (16.5)	1 (1.0)	<0.001

**Analgesic**	34 (33.0)	11 (10.7)	<0.001
**Antidepressant**	15 (14.6)	7 (6.8)	0.113
**Diabetes medication**	7 (6.8)	12 (11.7)	0.336
**Blood parameters**			
**Leukocytes, n/nl**	6.8 ± 1.8	8.3 ± 2.0	<0.001
**Erythrocytes, M/µl**	4.9 ± 0.4	4.7 ± 0.5	0.044
**Red cell distribution width (RDW), %**	13.1 ± 0.9	13.2 ± 1.0	0.436
**Hemoglobin, g/dl**	14.5 ± 1.4	14.3 ± 1.4	0.323
**Hematocrit, %**	43.8 ± 3.8	43.5 ± 3.7	0.511
**Mean corpuscular volume (MCV), fl**	90.3 ± 4.5	92.2 ± 5.3	0.007
**Mean corpuscular hemoglobin (MCH), pg**	29.9 ± 1.8	30.4 ± 2.1	0.111
**Mean cellular hemoglobin concentration (MCHC), g/dl**	33.1 ± 0.9	32.9 ± 1.1	0.192
**Thrombocytes, n/nl**	262.0 ± 64.6	288.1 ± 85.4	0.015
**HbA1c, %**	6.3 ± 1.5	6.6 ± 1.3	0.373
**C-reactive protein, mg/dl**	0.4 ± 0.6	0.4 ± 0.4	0.778
**Sodium, mmol/l**	140.9 ± 2.2	141.6 ± 2.5	0.050
**Potassium, mmol/l**	4.3 ± 0.4	4.5 ± 0.5	0.003
**Creatinine, mg/dl**	0.9 ± 0.2	1.0 ± 0.2	<0.001
**Urea, mg/dl**	30.9 ± 8.8	38.0 ± 9.8	<0.001
**eGFR, ml/min/1.73qm**	94.2 ± 15.2	86.8 ± 15.0	<0.001
**Uric acid, mg/dl**	5.8 ± 1.5	6.3 ± 1.5	0.011
**Triglycerides, mg/dl**	175.0 ± 116.8	156.1 ± 92.9	0.206
**Cholesterol, mg/dl**	217.9 ± 52.2	158.3 ± 37.9	<0.001
**HDL cholesterol, mg/dl**	56.1 ± 16.9	45.0 ± 10.4	<0.001
**LDL cholesterol, mg/dl**	141.2 ± 41.0	96.8 ± 29.1	<0.001
**LDL/HDL ratio**	2.7 ± 0.9	2.2 ± 0.7	<0.001
**Creatin kinase, U/l**	155.5 ± 163.8	140.7 ± 241.9	0.611
**Glutamate oxalacetate transaminase (GOT), U/l**	32.5 ± 14.5	31.1 ± 15.7	0.499
**Glutamate pyruvate transaminase (GPT), U/l**	40.0 ± 29.1	43.2 ± 28.5	0.424
**Gamma glutamyl transferase (gamma GT), U/l**	39.6 ± 28.1	49.9 ± 37.0	0.028
**Alkaline phosphatase, U/l**	80.0 ± 25.7	99.0 ± 37.5	<0.001
**Thyroid stimulating hormone (TSH), mIU/l**	1.8 ± 1.3	1.9 ± 1.4	0.691

Data presented as mean ± SD or n (%). Between-group comparison was performed using unpaired two-sided t-test or Chi-square test. § Determined by echocardiography (n=62 for PCS, n=92 for CAD). LVEF, left ventricular ejection fraction; PCI, percutaneous intervention; PCS, Post-Covid Syndrome; CAD, Coronary Artery Disease. Diseases/ symptoms with a prevalence < 5% are not reported.
