# Supplementary material for: Autonomic dysregulation in long-term patients suffering from Post-COVID-19 Syndrome assessed by heart rate variability
Source: Sci Rep. 2023 Sep 22;13:15814. doi: 10.1038/s41598-023-42615-y (PMC10516975; doi:10.1038/s41598-023-42615-y)
Supplement: Supplementary file 2 — Supplementary Table S2. [file 41598_2023_42615_MOESM2_ESM.pdf]

Supplementary Table S2: Assessment of physical fitness by cardiopulmonary exercise test (CPET)

	PCS (n=94)	CAD§ (n=68)	p-value
**Resting**			
Heart rate, beat·min^{-1}	90.1 ± 11.4	79.9 ± 11.9	<0.001
O_2 pulse, ml·beat^{-1}	6.6 ± 1.7	7.6 ± 2.4	<0.001
Ventilatory equivalent O_2 (VE/VO_2)	28.2 ± 5.0	32.1 ± 9.8	0.004
Ventilatory equivalent CO_2 (VE/VCO2)	34.3 ± 4.0	39.0 ± 10.5	<0.001
**Ventilatory threshold 1 (VT1)**			
Workload, % predicted	38.9 ± 16.4	44.9 ± 12.5	0.017
Heart rate, % predicted	64.4 ± 12.5	60.5 ± 8.6	0.034
O_2 pulse, % predicted	75.4 ± 18.4	85.7 ± 16.2	<0.001
VO_2, % predicted	49.1 ± 13.3	50.1 ± 12.8	0.645
Ventilatory equivalent O_2 (VE/VO_2), % predicted	79.8 ± 12.8	81.3 ± 13.0	0.481
Ventilatory equivalent CO_2 (VE/VCO2)	31.1 ± 4.1	31.1 ± 4.0	0.995
**Peak exercise**			
Respiratory exchange rate (RER)	1.05 ± 0.1	1.12 ± 0.1	<0.001
Workload, % predicted	71.1 ± 18.9	76.0 ± 21.6	0.135
Heart rate, % predicted	81.0 ± 11.5	74.9 ± 10.9	<0.001
O_2 pulse, % predicted	90.6 ± 17.7	101.4 ± 18.8	<0.001
VO_2, % predicted	72.7 ± 16.8	75.5 ± 16.0	0.290
Ventilatory equivalent O_2 (VE/VO_2), % predicted	99.6 ± 19.6	104.7 ± 17.8	0.104
Ventilatory equivalent CO_2 (VE/VCO_2)	32.9 ± 5.0	32.9 ± 4.0	0.942
**Respiratory minute ventilation (VE), % predicted**	58.0 ± 14.2	66.3 ± 16.0	<0.001
**Tidal volume (Vt), % predicted**	70.7 ± 19.4	71.1 ± 18.6	0.906
**Breathing frequency (Bf), % predicted**	54.3 ± 12.8	57.1 ± 13.1	0.193
**Breathing reserve (BR), %**	40.7 ± 17.5	39.8 ± 15.9	0.737

Data presented as mean ± SD. If indicated, results are presented as percent of predicted maximal values % predicted) for comparison, taking differences in sex and age between groups into account. §CAD patients after coronary artery bypass graft (CABG) were not included in the analysis. Between-group comparison was performed using unpaired two-sided t-test or Mann-Whitney U test. VO_2, oxygen uptake; PCS, Post-Covid Syndrome patients; CAD, Coronary Artery Disease patients.
